# Sorbic Acid‐Modified Soybean Oil: A Promising Biobased Molecular Platform for Sustainable Thermosetting Resins

**DOI:** 10.1002/cssc.202501346

**Published:** 2025-09-30

**Authors:** Selena Silvano, Adriano Vignali, Laura Boggioni, Fabio Bertini

**Affiliations:** ^1^ Institute of Chemical Sciences and Technologies “Giulio Natta” (SCITEC) National Research Council (CNR) Via Corti 12 20133 Milano Italy

**Keywords:** acrylic acid replacement, biobased materials, epoxidized soybean oil, sorbic acid, vegetable oil‐based thermosetting resins

## Abstract

In this article, the synthesis of a fully biobased molecular platform for thermosetting resins, namely sorbated epoxidized soybean oil (SESO), starting from sorbic acid (SA), and epoxidized soybean oil (ESO), is reported. The chemical structure of SESO is deeply characterized by spectroscopic and molecular analysis. The isothermal thermogravimetric analysis indicated that SA exhibited extremely lower volatility than acrylic acid (AA), which suggests that SA could be used as a green and safe alternative to AA overcoming issues related to AA volatility and toxicity that can be experienced during AA manipulation. Several SESO‐based thermosetting resins are obtained after the curing process performed by thermal treatment in the presence of a radical initiator. SESO shows complete curing after 6 h, faster than acrylated ESO (AESO), along with a good copolymerization ability with several reactive comonomers, such as styrene, myrcene, and pentaerythritol tetraacrylate, producing resins with similar mechanical (Young's modulus ranging from 6 to 1160 MPa) and thermal (glass transition temperature ranging from 17 to 61 °C) properties and superior biobased content to those AESO‐based. The successful synthesis of SESO offers the opportunity to use SA as an environmentally friendly alternative to AA in the synthesis of green thermosets.

## Graphical abstract

Sorbic acid and epoxidized soybean oil (ESO) are used for the first time to design and produce new green soybean oil‐based resins (SESO‐based resins). SESO‐based resins show superior biobased content and similar thermomechanical properties to those prepared by acrylated ESO.

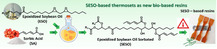

## 1 Introduction

The plastic market size is constantly increasing, especially that of thermosetting resin whose market size is expected to reach USD 97.02 billion by 2029, with an annual growth rate of 6.65% (forecast period 2021–2029).^[^ ^1^ ^]^ Thermosets are described as a class of polymers that upon curing reaction are irreversibly converted in 3D crosslinked structures.^[^ ^2^, ^3^ ^]^ They are of great interest for their widespread usage in coating, adhesive, composites, and electronic packaging fields, due to excellent dimensional stability, good chemical resistance, and outstanding thermal and mechanical properties.^[^ ^2^, ^4^, ^5^ ^]^ However, they mainly derived from nonrenewable petroleum‐based materials, such as bisphenol A, acrylic acid, styrene, and others.^[^ ^6^ ^]^ In recent years, the growing environmental and economic concerns as well as the uncertainty that accompanies limited petrochemical resources have driven the scientific community toward the use of more sustainable feedstocks with a lower impact on carbon emissions aiming to pursue the principles of green chemistry.^[^ ^6^, ^7^, ^8^, ^9^ ^]^ Among the different kinds of raw materials, vegetable oils represent an ideally renewable resources to obtain sustainable polymers with different structures and properties as they offer a large variability in terms of carbon skeleton, double bonds, and functionalities availability.^[^ ^6^, ^10^ ^]^ Soybean oil is suitable for polymer synthesis, since it is the most available among vegetable oils, with a global production over 60 million ton.^[^ ^11^ ^]^ However, the carbon–carbon double bonds belonging to vegetable oil structure present poor reactivity in typical polymerization limiting their direct use, thus chemical modification of oil (e.g., by epoxidation and/or acrylation) is necessary. Toward the synthesis of fully biobased thermosets, epoxidized soybean oil (ESO) in combination with curing agents derived from biomass, such as tri‐ and dicarboxylic acids (citric acid, itaconic acid, C4‐C10 and fatty‐derived diacids)^[^ ^10^, ^12^, ^13^, ^14^, ^15^, ^16^, ^17^, ^18^, ^19^ ^]^ or polyphenols (lignin and tannic acid),^[^ ^20^, ^21^, ^22^, ^23^, ^24^, ^25^ ^]^ was investigated. At the same time, acrylated ESO (AESO), due to the easiness of radical reaction of the acrylic group, has attracted notoriety in the last years.^[^ ^26^, ^27^, ^28^, ^29^ ^]^ Nonetheless, acrylic acid (AA), used in AESO preparation, is severely irritating and corrosive to the skin and the respiratory tract. Eye contact can result in serious and irreversible injury and high exposure can trigger pulmonary edema.^[^ ^30^ ^]^ To our knowledge, a few attempts have been made to replace AA with the aim of obtaining more environmentally friendly vegetable oil‐based resins. Li et al. used monomethyl itaconate, an itaconic acid derivative as an ecofriendly alternative to AA, obtaining resins with comparable tensile strength, lower modulus, and higher elongation at break than those formulated starting from AESO.^[^ ^30^ ^]^ Tang and Wu reported the modification of ESO with monomers containing two active C=C double bonds, that is, methallyl maleate or hydroxyethyl methacrylated maleate, achieving resins with increased tensile strength and modulus compared to those AESO‐based.^[^ ^31^, ^32^ ^]^

In this article, for the first time, the replacement of AA with sorbic acid (SA) for the synthesis of greener thermosets was proposed. SA is well‐known in the food industry since the early 1950s as food preservative for its inhibitory effects against a wide spectrum of yeasts, molds, and bacteria. It is directly extracted from nonedible berries of *Sorbus aucuparia*.^[^ ^33^, ^34^ ^]^ In the last years, the production of SA is growing very fast (up to reach 30 kt y^−1^), and since, the quantities extracted from the *S. aucuparia* are limited, the production of biosourced SA from bioethanol has been consolidated (through oxidation to acetaldehyde, trimerization to 2,4‐hexadienal, and final oxidation to SA).^[^ ^34^ ^]^ SA is a C6 carboxylic acid containing two conjugated carbon–carbon double bonds that can react with thermal or UV radical initiators to produce crosslinked structures. Here, fully biobased sorbated ESO (SESO) was synthetized from SA and ESO and characterized by thermal (thermogravimetric analysis [TGA]), spectroscopic (Nuclear Magnetic Resonance [NMR], fourier transformed infrared [FT‐IR]), molecular, and rheological analyses. Moreover, the volatility of SA was also investigated. Afterwards, SESO was used for thermosetting resins production by curing process in the presence of a thermal radical initiator. Since the flexible soybean oil skeleton, the use of reactive diluents, such as styrene^[^ ^35^, ^36^ ^]^ and divinylbenzene,^[^ ^37^, ^38^ ^]^ able to give a more crosslinked structure to vegetable oil resins, are explored in literature with the aim to obtain mechanical and thermal properties acceptable for end‐use sectors. For this reason, here, various resins were prepared in the presence of traditional comonomers, such styrene (STY), pentaerythritol tetraacrylate (PETRA), and myrcene (MY), as green alternative to conventional fossil‐based comonomers. The comonomer effect on mechanical, thermal, and dynamic‐mechanical properties of resins was studied. Moreover, a comparison, in terms of structure–properties relationship, between SESO‐based and AESO‐based resins was performed.

## 2 Experimental Section

### 2.1 Materials

Epoxidized soybean oil (ESO) was supplied by GreenSwitch (Ferrandina, Italy). SA (99%), triphenylphosphine (TPP) (99%), hydroquinone (99%), dichloromethane, and sodium hydrogen carbonate (NaHCO_3_) (99%) were purchased from ThermoFisher. Acrylated ESO (AESO), tert‐butyl peroxybenzoate (LuperoxP), myrcene (95%) (MY), styrene (99%) (STY), and pentaerythritol tetraacrylate (PETRA) were acquired from Sigma Aldrich. All reagents were used as received.

### 2.2 Synthesis of SESO

In the SESO preparation, 100 g of ESO, 28.4 g or 56.8 g of SA, 0.174 g of hydroquinone, and 1.72 g of TPP were charged into a round‐bottom flask equipped with a condenser and a magnetic stirrer. The mixture was stirred at 120 °C for 6.5 h under nitrogen atmosphere. During the work‐up procedure, the crude SESO was dissolved in dichloromethane and washed three times by saturated NaHCO_3_ aqueous solution. Then, the organic phase was dried with MgSO_4_, evaporated via rotary evaporation, and degassed under vacuum for 30 min before collection. Finally, a dark‐yellow viscous liquid was obtained. SESO was obtained by using 2.5 or 5 eq of SA, where 2.5 and 5 represent the mmol SA/mmol ESO ratio and named as SESO_2.5 or SESO_5, respectively. For the preparation of resins was exclusively used SESO_5.

### 2.3 Preparation of SESO and AESO Resins

In a typical procedure, the desired amount of SESO_5 or AESO (8.5 g) was put in a silicon mold (8 × 5.5 × 2.5 cm). Then, the proper amount of catalyst LuperoxP (0.0125% by weight) was added to the mold and well mixed with a spatula. The samples were treated in oven by applying the following optimized temperature programs: 2 h at 140 °C, 2 h at 160 °C, and 2 h at 180 °C for resins based on SESO_5 and 2 h at 140 °C, 2 h at 160 °C, and 4 h at 180 °C for resins based on AESO.^[^ ^6^ ^]^

### 2.4 Preparation of SESO‐Comonomer and AESO‐Comonomer Resins

In a becker, SESO_5 or AESO (8.5 g), catalyst (0.10–0.16 g), and comonomer (1.5−3.6 g) were manually stirred by using a spatula. Then, the mixture was put in a silicon mold (8 × 5.5 × 2.5 cm) and cured in oven by applying the following optimized temperature programs: 2 h at 140 °C, 2 h at 160 °C, and 2 h at 180 °C for SESO‐based resins and 2 h at 140 °C, 2 h at 160 °C, and 4 h at 180 °C for AESO‐based resins. The feed compositions for the preparation of resins are listed in **Table** **1**.

**Table 1:** Feed Composition of resins obtained from SESO_5 and AESO.

Sample	SESO_5 [g]	AESO [g]	MY [g]	STY [g]	PETRA [g]	LuperoxP [g]
R_SESO_	8.5	–	–	–	–	0.10
R_SESO‐MY_	8.5	–	1.5	–	–	0.13
R_SESO‐STY_	8.5	–	–	1.5	–	0.13
R_SESO‐PETRA_	8.5	–	–	–	1.5	0.13
R_SESO‐PETRA2_	8.5	–	–	–	3.6	0.16
R_AESO_	–	8.5	–	–	–	0.10
R_AESO‐MY_	–	8.5	1.5	–	–	0.13
R_AESO‐STY_	–	8.5	–	1.5	–	0.13
R_AESO‐PETRA_	–	8.5	–	–	1.5	0.13

### 2.5 Characterization

The volatility of SA and AA was evaluated by thermogravimetric analysis using a Perkin Elmer TGA 7 analyzer. Samples of about 5 mg were kept isothermally at 30 and 90 °C for 2 h in a nitrogen atmosphere.

^1^H‐NMR spectra of ESO, SESO, and AESO were obtained by dissolution of 15 mg of the sample in CDCl_3_ in a 5 mm tube. The spectra were acquired on a Bruker Avance II spectrometer operating at 500 MHz at room temperature. CDCl_3_ (standardization of chemical shifts of TMS at 7.24 ppm) was used as internal chemical shift reference. The applied conditions were the following: 5 mm probe, 90° pulse angle, acquisition time of 2.63 s, relaxation delay of 2 s, and 128 transients.

FT‐IR analyses were conducted on a Perkin Elmer Spectrum Two spectrometer in attenuated total reflectance mode within the range of wavenumber of 400–4000 cm^−1^, with a resolution of 4 cm^−1^ and 64 scans were collected for each sample. Spectra were normalized between 0 and 100 on transmittance value.

Gel permeation chromatography (GPC) analyses were performed on a Waters GPCV2000 system equipped with two PL Polypore columns, using THF with 0.05 wt% of antioxidant BHT as the mobile phase, at 35 °C with a 0.8 mL min^−1^ flow. The sample concentration was set at 2 mg mL^−1^ and the injection volume at 220 μL. The calibration of the GPC system was created by using narrow polystyrene standards in the 162–3 × 10^6^ g mol^−1^ range.

The gel content of the cured resins was determined in both ethyl acetate and chloroform. For the determination of gel fraction in ethyl acetate, a small amount of resin (≈200 mg) was placed into a paper filter and put in a closed tube in which 20 mL of ethyl acetate was added. The sample was soaked for 48 h, then, ethyl acetate was removed and the sample was dried in oven at 60 °C and weighed. For the determination of gel fraction in chloroform, the resin (200 mg) was put in a thumb filter and extracted under chloroform reflux by Soxhlet apparatus for 24 h. The sample was dried in oven at 60 °C and weighed. The gel fraction was calculated from the mass ratio of the resin after (m_1_) and before (m_0_) exposing it to solvent, as follows: Gel Content = m_1_/m_0_ × 100%.

Rheological experiments were performed at 25 °C with a TA Instruments AR 2000 rheometer using cone‐plate geometry (diameter 20 mm, angle 0.5°) in shear flow (40–1000 rad s^−1^) to measure the viscosity of ESO and SESO.

Dynamic mechanical analysis (DMA) was carried out on an Anton Paar MCR 702e instrument equipped with tension geometry. Rectangular‐shaped samples were tested setting a temperature ramp from −60 to 120 °C with a heating rate of 3 °C min^−1^, a single frequency oscillation of 1 Hz, and a strain amplitude of 0.07%.

TGA was carried out using a Perkin Elmer TGA 7 analyzer. The resins were tested from 50 to 750 °C at a scanning rate of 20 °C min^−1^ under nitrogen atmosphere.

The tensile mechanical tests were carried out on dumbbell specimens (overall length 75 mm, gauge length 25 mm, width of narrow section 4 mm) obtained by cutting the cured resins. The tests were conducted using a Zwick‐Roell Retro‐Line Z010 dynamometer with a load cell of 2.5 kN operating at a crosshead speed of 15 mm min^−1^ until break.

## 3 Results and Discussion

Here, the synthesis of SESO and the preparation of SESO‐based resins by thermal curing is presented. The synthesis of SESO and its characterization by FT‐IR, ^1^H‐NMR, and GPC was reported for the first time as well as the development (i.e., design, preparation, and characterization) of SESO‐based resins. All resins were studied by FT‐IR spectroscopy, thermal analysis, and mechanical and dynamic‐mechanical characterizations.

### 3.1 Volatility of SA and AA

A safe manipulation of resin's precursor can be important in perspective to scale up the production of SESO. For this purpose, the volatility of SA and AA was evaluated by isothermal TGA experiments at 30 and 90 °C under nitrogen flow, and the related thermograms are depicted in Figure S1 (Supporting Information). SA exhibited extremely low volatility at 30 °C and lower volatility than AA at 90 °C, due to its melting point of 135 °C.^[^ ^31^ ^]^ In detail, AA showed a high volatility at 30 °C with mass loss of 92% and volatilized completely within 15 min at 90 °C indicating that it is a volatile organic compound (VOC). The volatility of SA resulted much lower than AA as highlighted the absence of mass loss at 30 °C and a partial volatility at 90 °C after 2 h with mass loss of ≈50%. Hence, SA results to be a valid alternative to AA to solve the VOC emission issue in production of thermosetting resins.

### 3.2 Synthesis and Characterization of SESO

The synthesis of SESO, reported in Scheme S1 (Supporting Information), concerns the ring‐opening of ESO epoxy groups by SA through a solvent‐free process at lower temperature than SA melting point, performed in the presence of hydroquinone to preserve the C=C double bonds of SA and triphenyl phosphine as base. SESO was obtained using 2.5 or 5 equivalents of SA with respect to epoxy groups. The ring‐opening reaction was performed in bulk at 120 °C producing a dark‐orange slurry. The obtained product was purified by washing with NaHCO_3_ solution from unreacted SA obtaining a viscous fluid which was analyzed via FT‐IR and NMR spectroscopy.

**Figure 1:**
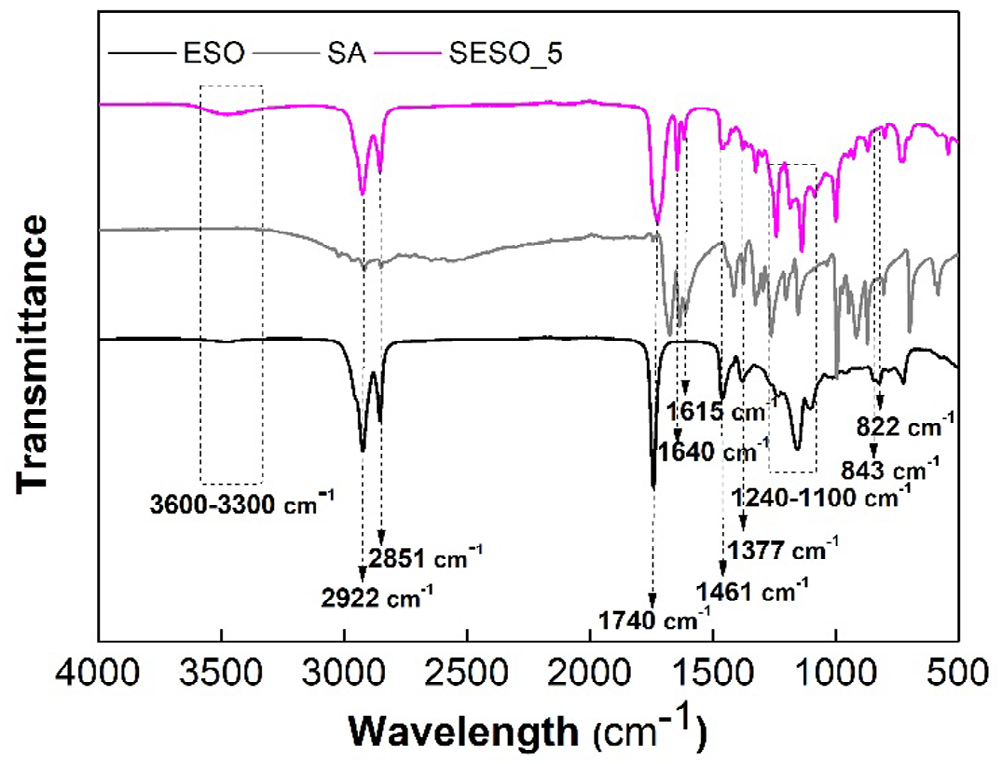
FT‐IR spectra of SESO_5 and monomers ESO and SA.

**Figure** **1** showed the FT‐IR spectra of SESO (as an example, SESO_5 was shown), compared with those of reagents ESO and SA. SESO spectrum showed the disappearance of epoxy groups characteristic bands (822 and 843 cm^−1^) together with the formation of a broad signal centered at about 3480 cm^−1^ ascribed to hydroxyl groups indicating the occurred ring‐opening reaction by SA. New signals associated to the SA presence appeared such as the twin bands at 1640–1615 cm^−1^ due to C=C group stretching vibrations. Moreover, characteristic peaks associated to ESO moiety were present such as the ester stretching bands C(=O)—O—C at about 1740 cm^−1^ and from 1240 to 1100 cm^−1^ and further bands at 2851−2922 cm^−1^ (methylene symmetric and antisymmetric stretch, respectively) and 1377−1461 cm^−1^ (methyl symmetric and antisymmetric deformations).^[^ ^39^, ^40^ ^]^

**Figure 2:**
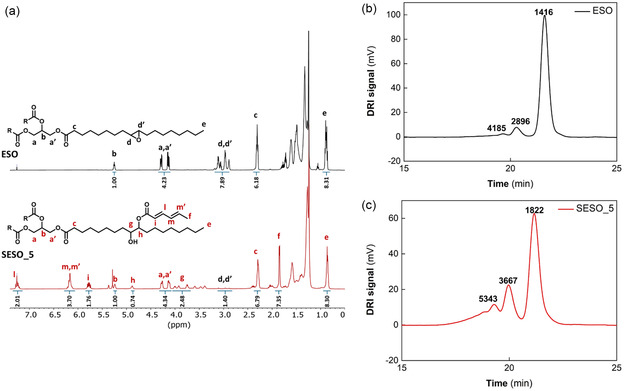
a) ^1^H‐NMR spectra of ESO and SESO_5, and GPC curves of b) ESO and c) SESO_5.

The ring‐opening reaction of epoxy groups by SA was also confirmed via ^1^H‐NMR analysis (**Figure** **2**a). The reduction of signals ascribed to the methine protons adjacent to epoxide group (signals d,d′ at 2.80–3.20 ppm) accompanied by the appearance of two signals: at 3.87–4.02 ppm (g) and at 4.80 ppm (h) attributable to methine protons in the α‐position of the —OH group (—CH—OH) and to the methine proton close to the sorbic group (ester) (—O=C—O—CH—) point out that a ring‐opening reaction occurred in SESO. New peaks due to the SA moiety were shown at 5.77–5.84 ppm (i), 7.23–7.31 ppm (l), 6.15–6.25 ppm (m,m′), and 1.88 ppm (f) attributed to C=C protons (α‐CH, β‐CH, γ‐CH to carbonyl group) and terminal methyl group, respectively.^[^ ^41^, ^42^ ^]^ Low intensity signals observed at about 3.5 ppm can be ascribed to ether bonds formation, probably, due to oligomerization side reactions that occurred during the sorbation process.^[^ ^43^ ^]^

The signals ascribed to the methine protons adjacent to epoxide group (d,d′) and (e) at 0.80–1.00 ppm were used as reference in the calculation of epoxy groups moles (*N*_Epox_) in ESO and SESO by Equation (1) (see also Figure S2–S4, Supporting Information).

(1) $$N_{\text{Epox }}=\frac{\left[\left(A_{\text{d,d′}}\right)\text{/2}\right]}{\left(A_{e}\text{/9}\right)}$$

*N*_Epox_ was equal to 4.27, 1.42, and 0.76 for ESO, SESO_2.5, and SESO_5, respectively. Thus, the yield of ring‐opening reaction was estimated by Equation (2) and resulted to be equal to 66.7% for SESO_2.5 and 82.2 % for SESO_5.

(2) $$\text{Yield = 100}-\left[\left(\frac{N_{\text{Epox}}\text{ in SESO}}{N_{\text{Epox}}\text{ in ESO}}\right)\text{×100}\right]$$

The SA moles (*N*_SA_) grafted to ESO and the moles of C=C functionality in SESO were calculated according to Equation (3) and (4), respectively, by using as reference the peak at 0.80–1.00 ppm (e) (Figure S3 and S4, Supporting Information).^[^ ^31^ ^]^

(3) $$N_{\text{SA}}=\frac{\left[(A_{l}+A_{i}+A_{m,m\prime })/4\right]}{\left(A_{e}/9\right)}$$

(4) $$N_{C=C}=\frac{\left[\left(A_{l}+A_{i}+A_{m,m\prime }\right)/4\right]}{\left(A_{e}/9\right)}*2$$

*N*_SA_ and *N*_C=C_ in SESO were equal to 1.6 and 3.2 for SESO_2.5, and 2.0 and 4.0 for SESO_5. Although, in commercial AESO the AA moles grafted to ESO was higher than SA moles present in SESO_5 (2.8 vs 2.0) (Figure S5, Supporting Information), the C=C functionality of SESO_5 resulted higher than AESO because SA contains two double bonds C=C per molecule. An in‐depth molecular characterization was performed for ESO and SESO_5 by GPC and by the polystyrene calibration curve, the molar masses were calculated (Figure 2b,c).

ESO showed three molar mass populations, where the main fraction (87%) with peak molar mass close to 1400 g mol^−1^ was accompanied by other two minor fractions with molar mass twice (≈8%) and three times (≈5%) higher. The chromatogram of SESO_5 showed a similar profile with three distinct peaks shifted to shorter retention times because of the enhanced molar masses.

The main peak accounted for about 60% and presented a molar mass close to 1800 g mol^−1^. Therefore, after the sorbation, the amount of populations with higher molar mass increased to ≈40%. The increase in the high molar mass fraction for SESO_5 can be ascribed to oligomerization side reactions that occurred during the reaction,^[^ ^32^ ^]^ as highlighted from NMR analysis.

The viscosity of ESO and SESO_5 was measured by rotational rheometry and the flow curves are shown in Figure S6 (Supporting Information). Both samples exhibited Newtonian behavior in investigated range of shear rates and according to molecular characterization, SESO_5 showed a markedly higher viscosity than ESO. In detail, the values of viscosity of 0.45 and 110 Pa s were determined at 25 °C for ESO and SESO_5, respectively.

The thermal stability of SESO_5 and AESO was evaluated by means of TGA and the thermograms are reported in Figure S7a (Supporting Information). The samples exhibited a very similar thermal behavior with an onset degradation temperature close to 340 °C, measured in correspondence of 5% mass loss, and a single well‐defined decomposition event between 340 and 500 °C centered at 426–428 °C as revealed by derivative thermogravimetry (DTG) curves (Figure S7b, Supporting Information).

### 3.3 Preparation and Characterization of Resins

SESO‐based resins were obtained exclusively from SESO_5 because it contains about 25% more C=C functionality compared to SESO_2.5, and thus, could be more reactive toward crosslinking process. SESO_5 was cured in presence of radical initiator LuperoxP without or with some comonomers, such as styrene, myrcene, and pentaerythritol tetraacrylate, used at 15 wt% (Figure S8, Supporting Information). The obtained resins were named as R_SESO_, R_SESO‐MY_, R_SESO‐STY_, and R_SESO‐PETRA_, respectively. Moreover, the addition of 30 wt% PETRA comonomer was explored obtaining the resin noted as R_SESO‐PETRA2_. For comparison, AESO‐based resins were also prepared. The curing of SESO‐based thermosets was completed after 6 h in the temperature range from 140 to 180 °C, whereas the AESO‐based resins required further 2 h at 180 °C, highlighting the more reactivity of SESO in the radical polymerization and the subsequent formation of crosslinked materials.

All resins looked like yellow or dark orange solids (Figure S9, Supporting Information) and were insoluble in common organic solvent. The gel content, determined by weighting method, is commonly used for the evaluation of crosslinked structures in thermosetting resins. In fact, the crosslinked fraction can only be swelled, while that uncrosslinked can be dissolved in suitable solvents.^[^ ^44^ ^]^ The gel content, performed in ethyl acetate and chloroform, highlighted an insoluble fraction ranging from 93 to 96 wt% indicating a high crosslinking degree for all investigated resins (Table S1 in Supporting Information). In particular, resins prepared with MY exhibit the lowest gel content values while those containing PETRA the highest ones. Therefore, the use of PETRA comonomer, characterized by a high number of reactive vinylic groups, enhances the degree of crosslinking in thermosetting resins.

The biobased content of samples was evaluated by the following Equation (5)^[^ ^6^ ^]^ and reported in **Table** **2**.

(5) $$\text{Biobased content}=\frac{\sum \left(\text{biobased carbon content }\times \text{ weight fraction of component in resin}\right)}{\sum \left(\text{total carbon content }\times \text{ weight fraction of component in resin}\right)}\times 100$$

where “biobased carbon content” and “total carbon content” are, respectively, the wt% of biobased carbon and the total wt% of carbon within SESO or AESO and comonomer. The “weight fraction of component in resin” is referred to the weight fraction of SESO or AESO and comonomer in the resin formulation.

**Table 2:** Biobased content and DMA properties of resins.

Sample	Biobased content [%]	DMA properties			
		*T*_g_ [°C]a)	*E*′_−50°C_ [MPa]b)	*E*'_*T*g+50°C_ [MPa]b)	*ν* [mol m^−3^]c)
R_SESO_	100	33	1269	11	1235
R_SESO‐MY_	100	17	1860	7	825
R_SESO‐STY_	81	43	1692	9	986
R_SESO‐PETRA_	88	52	1885	58	6201
R_SESO‐PETRA2_	74	61	2693	243	25 370
R_AESO_	82	26	1528	18	2074
R_AESO‐MY_	86	17	1156	7	825
R_AESO‐STY_	66	54	1760	13	1382
R_AESO‐PETRA_	72	53	2175	71	7570

a) Glass transition temperature determined by DMA.

b) Storage modulus measured respectively at −50 °C and *T*_g_ + 50 °C.

c) Crosslink density calculated using Equation (6).

The total carbon content in ESO is 72 wt% and moves to 70 and 67 wt% in SESO and AESO, respectively, whereas their biobased carbon content is equal to 70 and 55 wt%, respectively, due to sorbation and acrylation modification. It is worth noting that the samples R_SESO_ and R_SESO‐MY_ are completely biobased (biobased content = 100%). The incorporation of fossil‐based comonomers, such as styrene and pentaerythritol tetraacrylate, slightly penalizes the biobased content of SESO‐based resins, nevertheless it remains between 74% and 88%. However, SESO‐based resins always present a biobased content markedly superior to their AESO‐based counterparts (Table 2).

**Figure 3:**
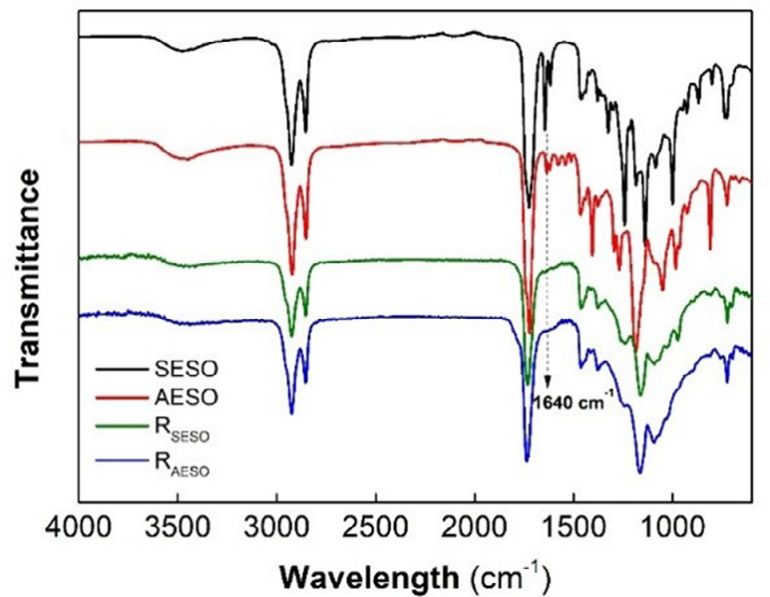
FT‐IR of R_SESO_ and R_AESO_ vs uncured SESO and AESO.

FT‐IR spectra of resins showed that after curing, the signals attributable to C=C stretching vibrations (1640 cm^−1^) belonging to AA and SA moieties disappeared (**Figure** **3**).^[^ ^45^ ^]^ The lacking in C=C signals was attributable to their involvement in the crosslinking process. The same behavior, that is, the involvement of C=C double bond in network formation, was found in the spectra of resins obtained in presence of reactive comonomers (Figure S10 and S11, Supporting Information).

**Figure 4:**
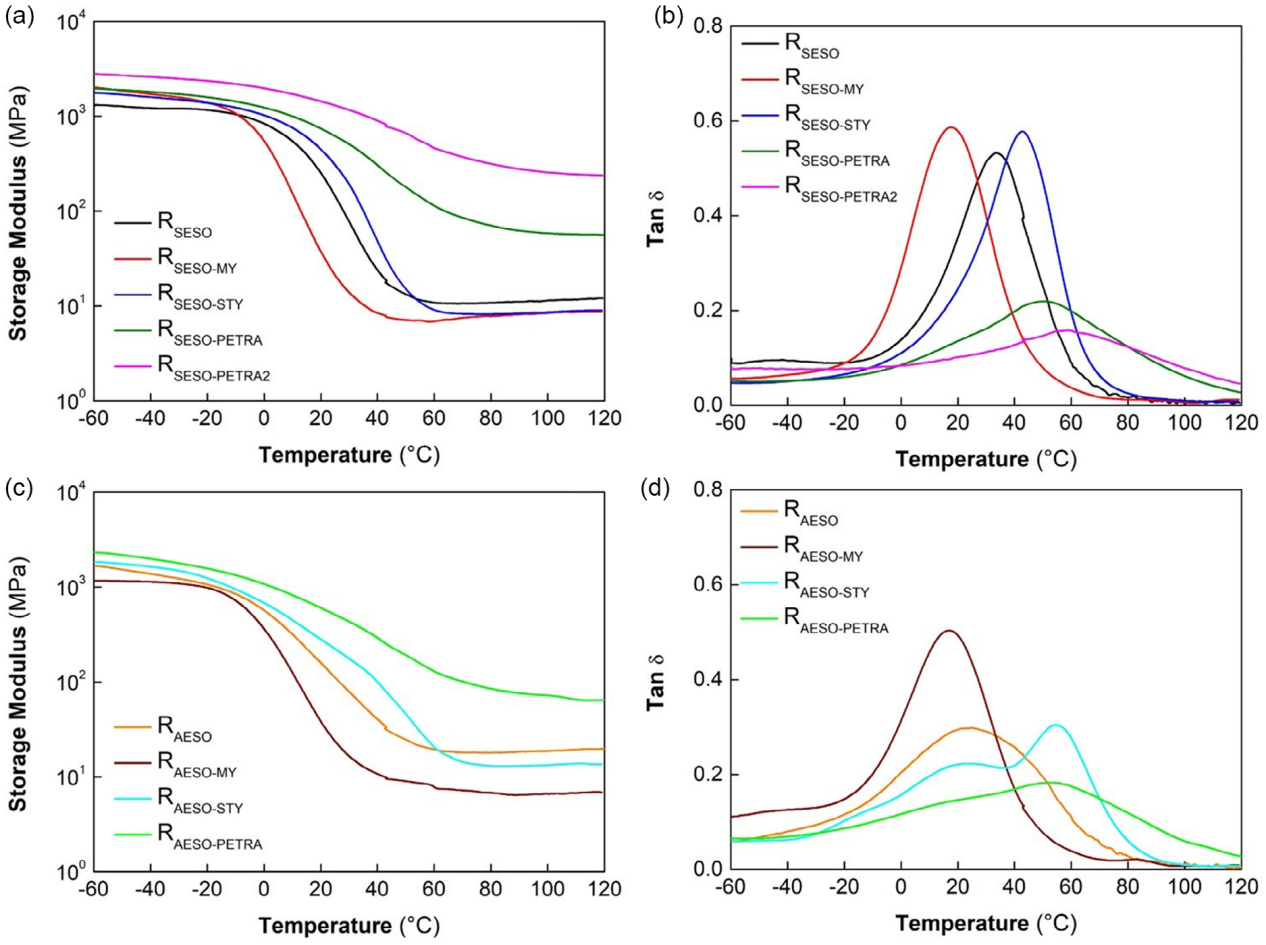
DMA curves of resins based on a,b) SESO and c,d) AESO.

The resins were tested by DMA and the results were shown in **Figure** **4** and Table 2. The samples were compared in terms of variation of storage modulus *(E′)* and loss factor (*tan δ*) as a function of temperature. Table 2 summarizes the glass transition temperature (*T*_g_), determined as the maximum peak temperature of *tan δ*, the storage moduli of resins measured in glassy region (*E′*_*–*50 °C_), and rubbery region (*E′*_*T*g + 50 °C_) at −50 °C and *T*_g_ + 50 °C, respectively, and the crosslink density of the cured samples determined using Flory theory of rubber elasticity following Equation (6).^[^ ^46^ ^]^

(6)

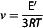

where *E′* is the storage modulus of the sample in the rubbery plateau region at *T* =* T*_g_ + 50 in Kelvin and *R* is the gas constant (8.31 J mol^−1 ^K^−1^).

As can be seen in Table 2, the SESO‐ and AESO‐based resins with the same composition generally exhibited similar *T*_g_. In detail, R_SESO_ and R_AESO_ showed *T*_g_ of 33 and 26 °C, respectively. Moreover, R_SESO_ presented in the whole analyzed temperature range a stiffness slightly lower than R_AESO_, thus, the SESO‐based resin crosslink density is lower than AESO‐based sample. In general, the lower crosslink density of SESO‐based resins can be ascribed to not complete availability of C=C double bond to network formation. Some studies demonstrated the formation of SA 1,4‐adduct and the occurring of cycloadditions as side reaction.^[^ ^47^, ^48^ ^]^ Furthermore, during radical polymerization, the presence of allylic hydrogens next to the double bonds in SA moiety could lead to chain transfer side reaction and polymer chain termination.^[^ ^49^ ^]^ The presence of MY leads to resins characterized by reduced crosslink density and *T*_g_ (17 °C) due to the presence of allylic hydrogens, which affects the crosslinking reaction, the high flexibility, and molecular mobility of crosslinked structure. As STY is added in the resins, higher *T*_g_ values was measured both when the reaction was conducted with SESO and AESO. This can be rationalized, on the basis of STY structure presenting a rigid aromatic unit that enhanced the rigidity of the chain segments. Both styrene containing resins exhibited lower crosslinking density than R_SESO_ and R_AESO_ which can be ascribed to chain extender ability of STY leading to decreased crosslinking degree.^[^ ^50^ ^]^ The addition of PETRA led to considerable increase in *T*_g_ (>50 °C), storage modulus, and crosslink density of resins, because of the highly available vinylic group arranged as “four arm‐star shaped” that generated a rigid network during the curing process. This behavior is further highlighted, both for SESO‐ and AESO‐based resins, by the low intensity of *tan δ* that suggests reduced mobility of chain segments. In particular, R_SESO‐PETRA2_ prepared with a double content of PETRA, showed a *T*_g_ of 61 °C and the ability to create a denser crosslinked structure than the other samples, due to the higher amount of vinylic groups available for the crosslinking process.

**Figure 5:**
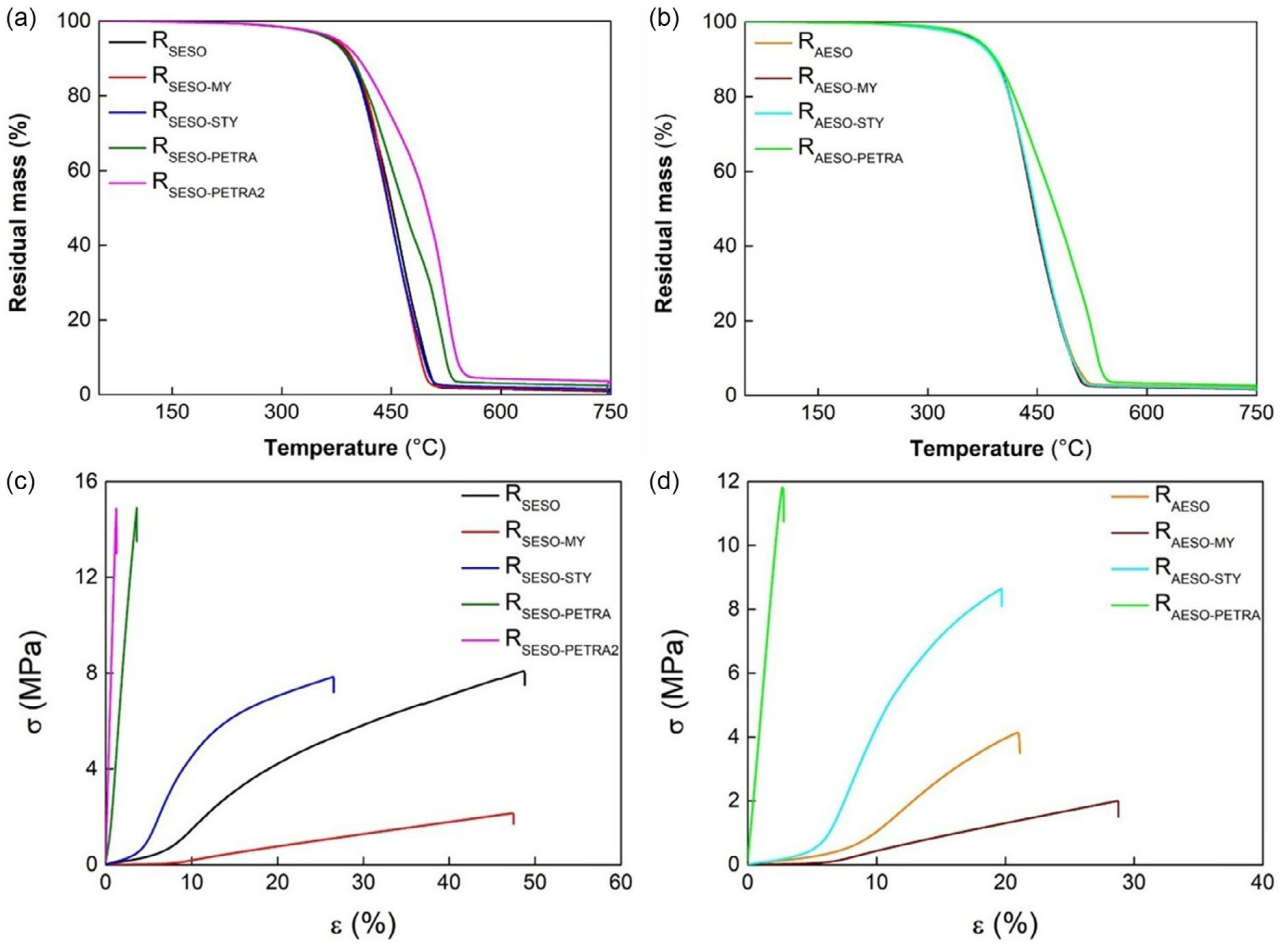
TGA thermograms of resins based on a) SESO and b) AESO, and tensile curves of resins based on c) SESO and d) AESO.

The thermal degradation of the resins was evaluated by TGA carried out under inert atmosphere (**Figure** **5**a,b and S12, Supporting Information). **Table** **3** summarizes the characteristic temperatures of degradation process, that is, *T*_5%_, a measurement of the decomposition onset taken at 5% mass loss, *T*_50%_ the midpoint of the degradation process, *T*_max_ the maximum rate degradation temperature, and *R* the fraction of residual material at 750 °C. In general, the SESO‐based resins showed high thermal stability with a decomposition pattern consistent with other soybean oil‐based thermosetting resins.^[^ ^6^, ^16^, ^32^ ^]^ The initial phase of the thermal degradation process is similar for all SESO‐based resins, as well shown by the comparable *T*_5%_ values spanning from 367 to 375 °C. As the degradation process proceeds, the thermal behavior of R_SESO_, R_SESO‐MY_, and R_SESO‐STY_ resins remains substantially similar, as well evidenced by the *T*_50%_ values going from 445 to 449 °C, while the resins with PETRA continue to degrade at a much slower rate (Figure 5a). Moreover, unlike R_SESO_, R_SESO‐MY_, and R_SESO‐STY_, which exhibit a single peak of degradation centered at ≈450 °C, the DTG trace of both PETRA containing resins is characterized by overlapping decomposition events (Figure S12a, Supporting Information). The multistage decomposition with a main peak centered at 514 °C (523 °C for R_SESO‐PETRA2_) and a pronounced shoulder at about 440 °C, indicates that the decomposition products are released in steps, which reflect the different involved mechanisms. The increased thermal stability for the resins containing PETRA was attributed to the high crosslink density and chemical structure of cured network. A very similar behavior was observed for AESO‐based resins (Figure 5b and S12b, Supporting Information); in particular the addition of PETRA as comonomer to formulation, contributed to improve the thermal stability of AESO‐based resin increasing the midpoint and the maximum rate decomposition temperatures. The residue amount at 750 °C was between 1 and 3.6 wt%, with the higher content for the resins prepared with PETRA as comonomer. Under air exposure at 750 °C, the carbonaceous residue was oxidized and disappeared completely. The mechanical behavior of resins was evaluated by uniaxial tensile test and the recorded stress−strain curves are shown in Figure 5c,d. Tensile properties, such as Young's modulus (*E*), maximum strength (*σ*_max_), and elongation at break (*ε*), were averaged over five samples at least and reported with their standard deviations in Table 3.

**Table 3:** Thermal and mechanical properties of resins.

Sample	Mechanical properties		Thermal properties				
	*E* [MPa]a)	*σ*_max_ [MPa]b)	*ε* [%]c)	*T*_5%_ [°C]d)	*T*_50%_ [°C]e)	*T*_max_ [°C]f)	*R* [%]g)
R_SESO_	30 ± 4	7.4 ± 0.7	50 ± 3	369	448	452	1.0
R_SESO‐MY_	6 ± 1	2.3 ± 0.4	47 ± 5	370	447	442	1.2
R_SESO‐STY_	68 ± 9	7.7 ± 0.9	27 ± 4	367	447	448	1.4
R_SESO‐PETRA_	432 ± 45	14.8 ± 3.8	4.3 ± 0.9	367	468	(s 440) 514	2.5
R_SESO‐PETRA2_	1160 ± 89	14.5 ± 1.6	1.3 ± 0.1	375	499	(s 440) 523	3.6
R_AESO_	36 ± 2	4.3 ± 0.6	26 ± 3	369	445	440	2.0
R_AESO‐MY_	9 ± 1	2.3 ± 0.3	35 ± 2	366	445	440	1.7
R_AESO‐STY_	91 ± 12	8.3 ± 1.3	20 ± 1	365	447	447	1.9
R_AESO‐PETRA_	479 ± 38	11.8 ± 1.2	2.7 ± 0.5	370	475	(s 440) 523	3.2

a) *E*: Young's modulus.

b) *σ*_max_: maximum tensile strength.

c) *ε*: elongation at break.

d) Temperature of 5% mass loss.

e) Temperature of 50% mass loss.

f) Temperature of maximum rate of mass loss.

g) Residue yield at 750 °C in nitrogen atmosphere.

R_SESO_ showed mechanical properties similar, in terms of Young's modulus (30 MPa), and even higher than R_AESO_ as regards maximum tensile strength (7.4 MPa) and elongation at break (50%). The resins containing STY exhibited enhanced Young's modulus, unchanged *σ*_max_, and decreased *ε* compared to R_SESO_ and R_AESO_. For both SESO‐ and AESO‐based resins, *σ*_max_, and *E* were strongly improved by incorporating the reactive comonomer PETRA due to the high C=C availability and crosslink density. This result is further highlighted as increasing the PETRA amount. In detail, a double content of this comonomer led to an increase in the *E* value of about 170%. The presence of the comonomer MY, instead, leads to a decrease in stiffness and mechanical strength while maintaining *ε* similar to that measured for R_SESO_ and R_AESO_, in agreement with DMA data.

## 4 Conclusion

Here, it was proposed the use of SA as possible alternative to AA in the preparation of thermosetting soybean oil‐based resins since SA address to volatility, toxicity, and fossil‐derivation issues correlated to the use of AA. For the first time, fully biobased SESO and its resins were synthetized and characterized by spectroscopic, rheological, thermal, and DMA.

SESO was obtained in mild reaction conditions achieving the ring‐opening yield of the epoxide groups belonging to ESO over the 80% and leading 2 sorbate groups within the triglyceride molecule, thus, SESO showed higher amount of C=C double bonds (4 mol per SESO molecule) than commercial AESO (2.8 mol per AESO molecule). In order to highlight the great potential of SESO for producing sustainable thermosets alternative to traditional resins based on acrylic acid, SESO was then thermal cured without or with a comonomer, such as myrcene, styrene, and pentaerythritol tetraacrylate. In general, the resins prepared starting from SESO exhibited a biobased content of 75%−100% superior to their AESO‐based counterparts. In particular, the samples R_SESO_ and R_SESO‐MY_ resulted fully biobased thanks to the use of SA and terpenic comonomer. The addition of PETRA led to highly crosslinked network resins due to its structure containing many reactive vinylic groups, while maintaining a high biobased content (88%) due to the low amount of comonomer (15 wt%). The dynamic‐mechanical analysis showed that the thermosets based on SESO owned a comparable behavior to AESO resins in terms of *T*_g_, storage modulus, and crosslinking density. Moreover, SESO‐based resins exhibited tunable thermal and mechanical performances depending on different comonomers used. Overall, the data confirmed the possibility to manufacture thermosetting resins from SA with very similar properties to AESO‐based resins but more sustainable avoiding the issues correlated to the use of AA in the modification of ESO.

## Conflict of Interest

The authors declare no conflict of interest.

## Supporting information

Supplementary Material

## Data Availability

The data that support the findings of this study are available in the supporting information of this article.
