# Supplementary material for: Sorbic Acid‐Modified Soybean Oil: A Promising Biobased Molecular Platform for Sustainable Thermosetting Resins
Source: ChemSusChem. 2025 Sep 30;18(22):e202501346. doi: 10.1002/cssc.202501346 (PMC12642972; doi:10.1002/cssc.202501346)
Supplement: Supplementary file 1 — Supplementary Material [file CSSC-18-e202501346-s001.pdf]

Supporting Information

Sorbic Acid-modified Soybean Oil: A Promising Bio-Based Molecular Platform for Sustainable Thermosetting Resins

Selena Silvano, Adriano Vignali, Laura Boggioni, and Fabio Bertini

Figure S1. Isothermal TGA curves of sorbic acid and acrylic acid at 30 and 90 °C 2

Scheme S1. SESO synthesis starting from ESO 2

Figure S2. ^{1}H-NMR spectrum of ESO (500MHz, $CDCl_3$) 3

Figure S3. ^{1}H-NMR spectrum of SESO_2.5 (500MHz, $CDCl_3$) 4

Figure S4. ^{1}H-NMR spectrum of SESO_5 (500MHz, $CDCl_3$) 4

Figure S5. ^{1}H-NMR spectrum of AESO (500MHz, $CDCl_3$) 5

Figure S6. Flow curves of ESO and SESO_5.. 5

Figure S7. (a) TGA and (b) DTG curves of SESO_5 and AESO.. 6

Table S1. Gel fraction test of SESO and AESO-based resins in ethyl acetate (EtOAc) and chloroform ($CHCl_3$).. 6

Figure S8. Chemical structure of comonomers used for the preparation of resins.. 7

Figure S9. Resins obtained from SESO.. 7

Figure S10. FTIR of resins prepared from AESO: (a) $R_{AESO\text{-}MY}$, (b) $R_{AESO\text{-}STY}$ and (c) $R_{AESO\text{-}PETRA}$.. 8

Figure S11. FTIR of resins prepared from SESO: (a) $R_{SESO\text{-}MY}$, (b) $R_{SESO\text{-}STY}$ and (c) $R_{SESO\text{-}PETRA}$ 9

Figure S12. DTG curves of resins based on (a) SESO and (b) AESO 10

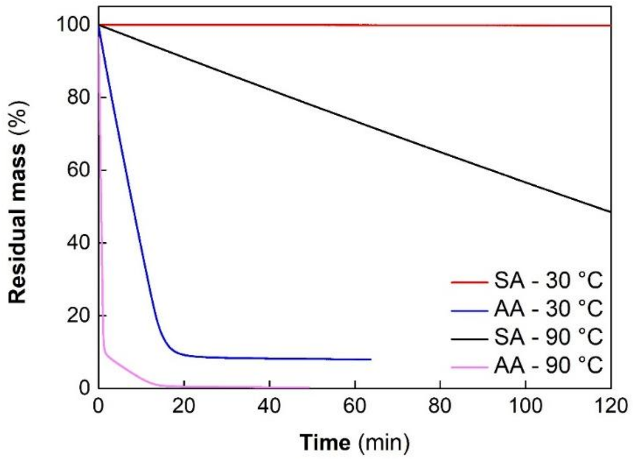

Figure S1. Isothermal TGA curves of sorbic acid and acrylic acid at 30 and 90 °C

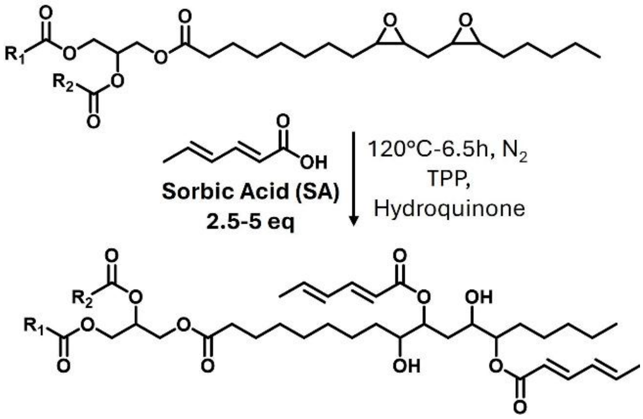

Scheme S1. SESO synthesis starting from ESO

ESO

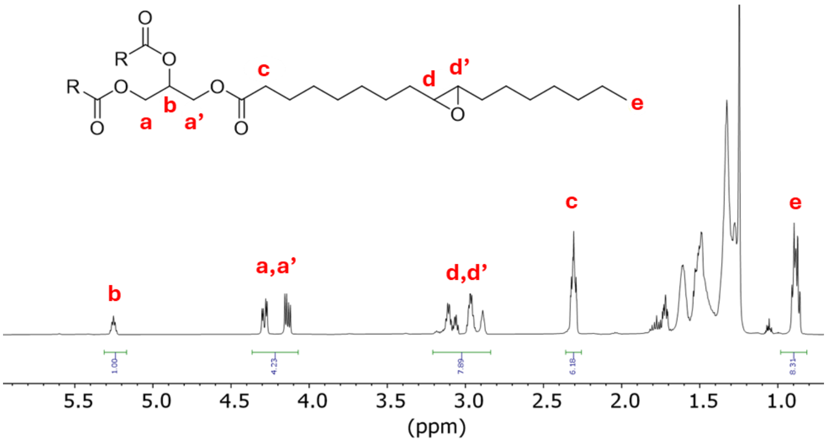

Figure S2. ^{1}H-NMR spectrum of ESO (500MHz, $CDCl_3$)

$$N_{Epox\ ESO} = \frac{(A_{d,d'}/2)}{(A_e/9)} = \frac{7.89/2}{(8.31/9)} = 4.27$$

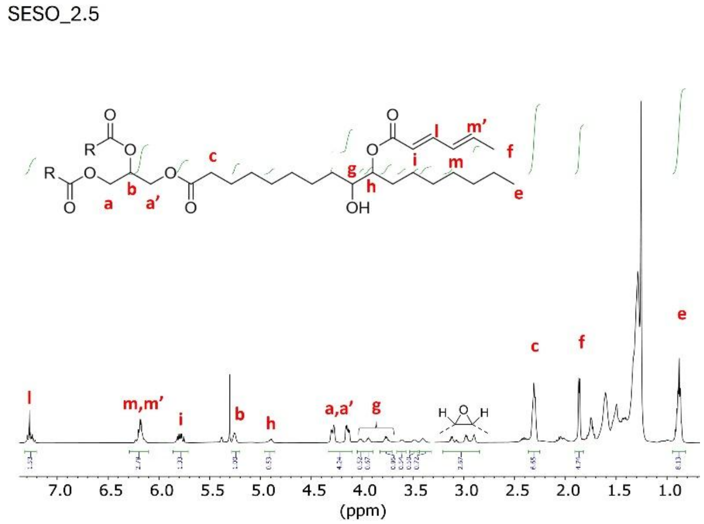

Figure S3. ^{1}H-NMR spectrum of SESO_2.5 (500MHz, $CDCl_3$)

$$N_{C=C\ SESO\_2.5} = \frac{[(A_l + A_i + A_{m,m'})/4]}{(A_e/9)} * 2 = \frac{[(1.53 + 1.33 + 2.79)/4]}{(8.13/9)} * 2 = 1.56 * 2 = 3.13$$

$$N_{Epox\ SESO\_2.5} = \frac{(A_{d,d'}/2)}{(A_e/9)} = \frac{(2.57/2)}{(8.13/9)} = 1.42$$

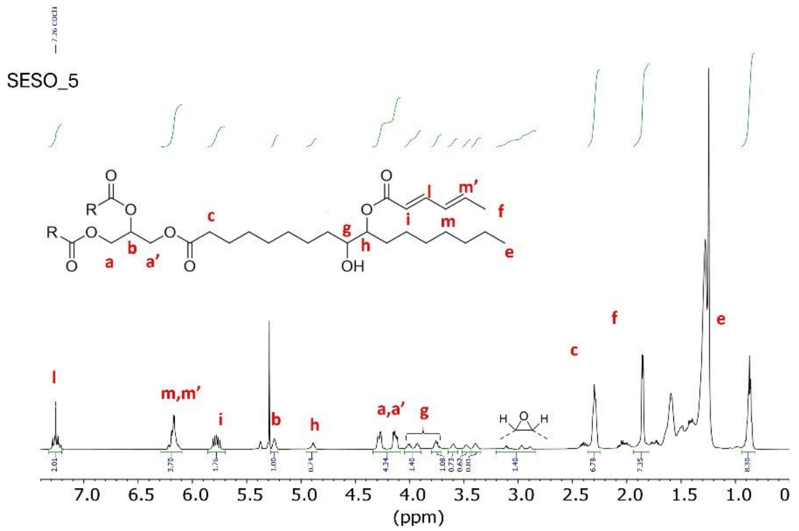

Figure S4. ^{1}H-NMR spectrum of SESO_5 (500MHz, $CDCl_3$)

$$N_{C=C\ SESO\_5} = \frac{[(A_l + A_i + A_{m,m'})/4]}{(A_e/9)} * 2 = \frac{[(2.01 + 1.76 + 3.70)/4]}{(8.30/9)} * 2 = 2.025 * 2 = 4.05$$

$$N_{Epox\ SESO\_5} = \frac{(A_{d,d'}/2)}{(A_e/9)} = \frac{(1.40/2)}{(8.30/9)} = 0.76$$

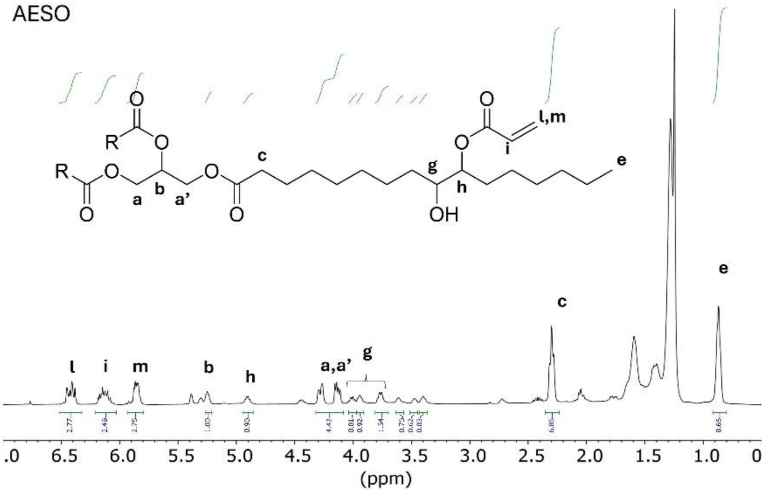

Figure S5. ^{1}H-NMR spectrum of AESO (500MHz, $CDCl_3$)

$$N_{C=C\ AESO} = \frac{[(A_l + A_i + A_m)/3]}{(A_e/9)} = \frac{[(2.77 + 2.49 + 2.75)/3]}{(8.66/9)} = 2.78$$

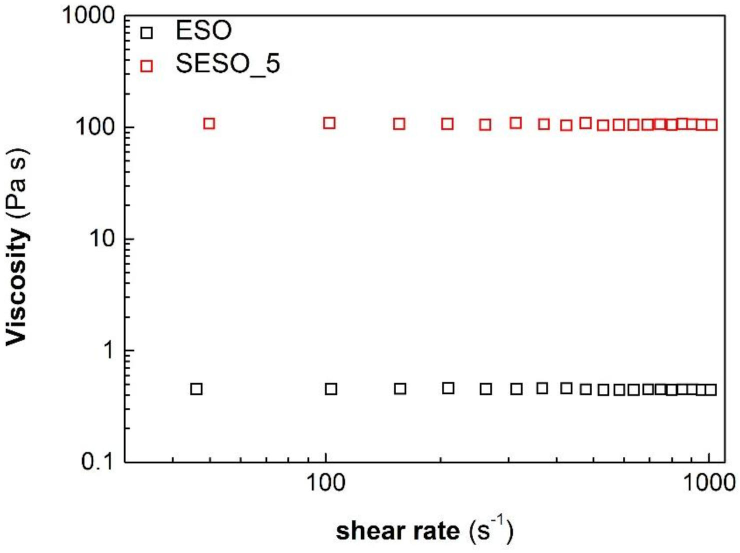

Figure S6. Flow curves of ESO and SESO_5

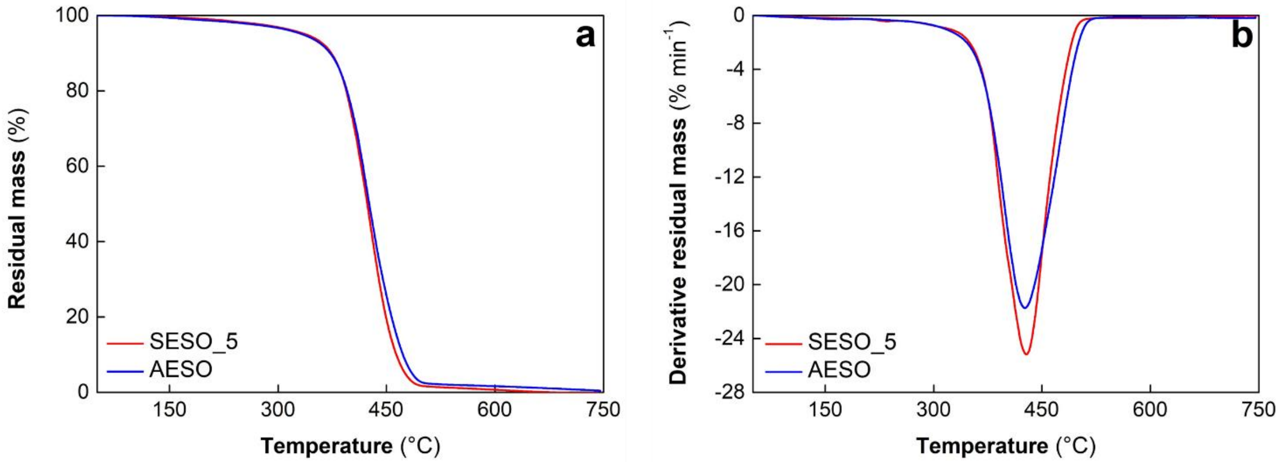

Figure S7. (a) TGA and (b) DTG curves of SESO_5 and AESO

Samples	Gel fraction (wt. %)	
	EtOAc	$CHCl_3$
R_{SESO}	95.1	95.0
$R_{SESO\text{-}MY}$	94.0	93.8
$R_{SESO\text{-}STY}$	95.4	94.9
$R_{SESO\text{-}PETRA}$	95.2	95.1
$R_{SESO\text{-}PETRA2}$	95.3	95.0
R_{AESO}	94.7	94.5
$R_{AESO\text{-}MY}$	94.3	94.1
$R_{AESO\text{-}STY}$	95.2	95.0
$R_{AESO\text{-}PETRA}$	95.6	95.3

Table S1. Gel fraction test of SESO and AESO-based resins in ethyl acetate (EtOAc) and chloroform ($CHCl_3$)

Myrcene	Styrene	Pentaerythritol tetraacrylate
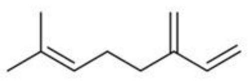	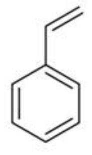	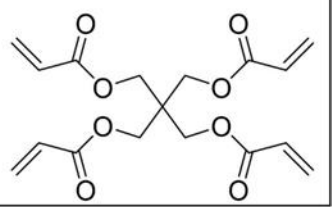

Figure S8. Chemical structure of comonomers used for the preparation of resins

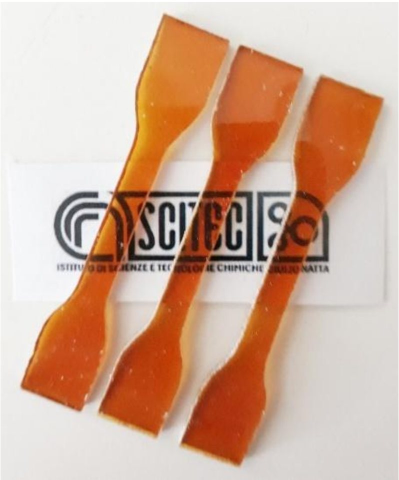

Figure S9. Resins obtained from SESO

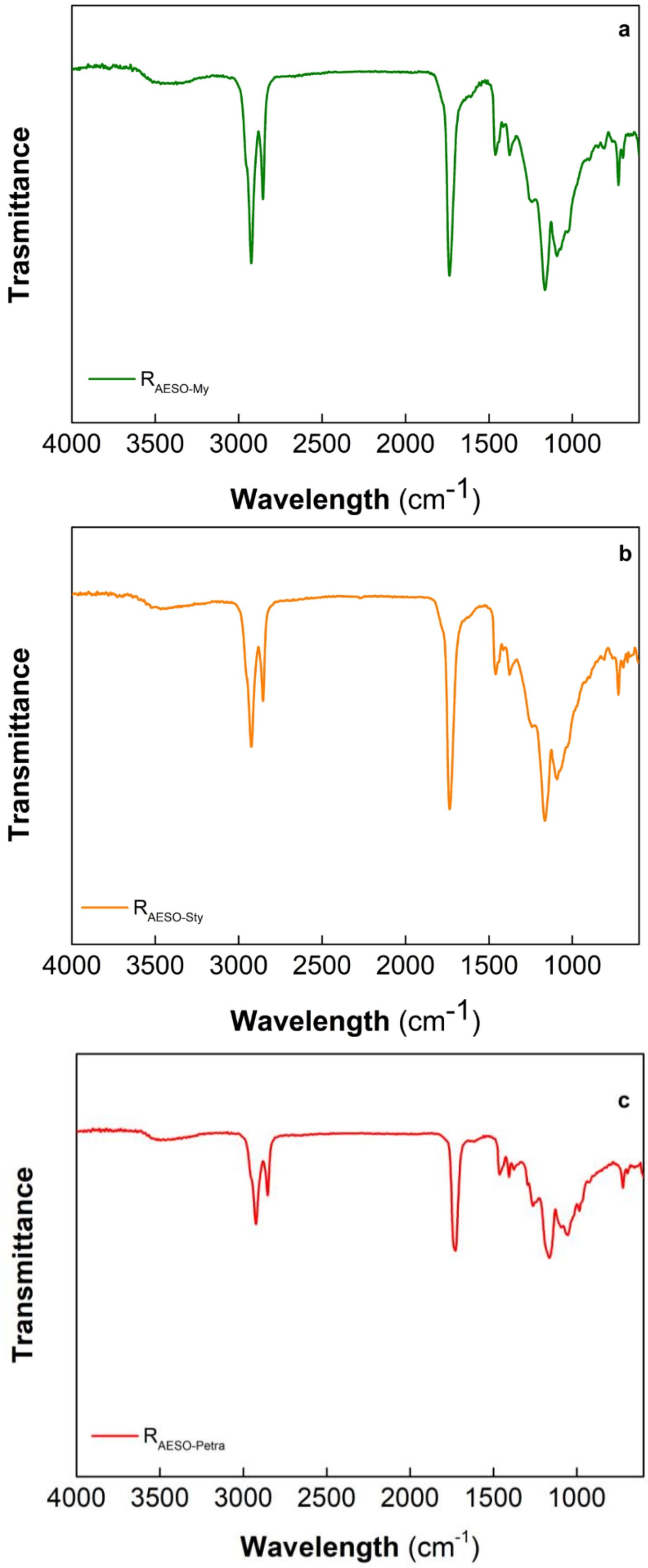

Figure S10. FTIR of resins prepared from AESO: (a) $R_{AESO\text{-}MY}$, (b) $R_{AESO\text{-}STY}$ and (c) $R_{AESO\text{-}PETRA}$

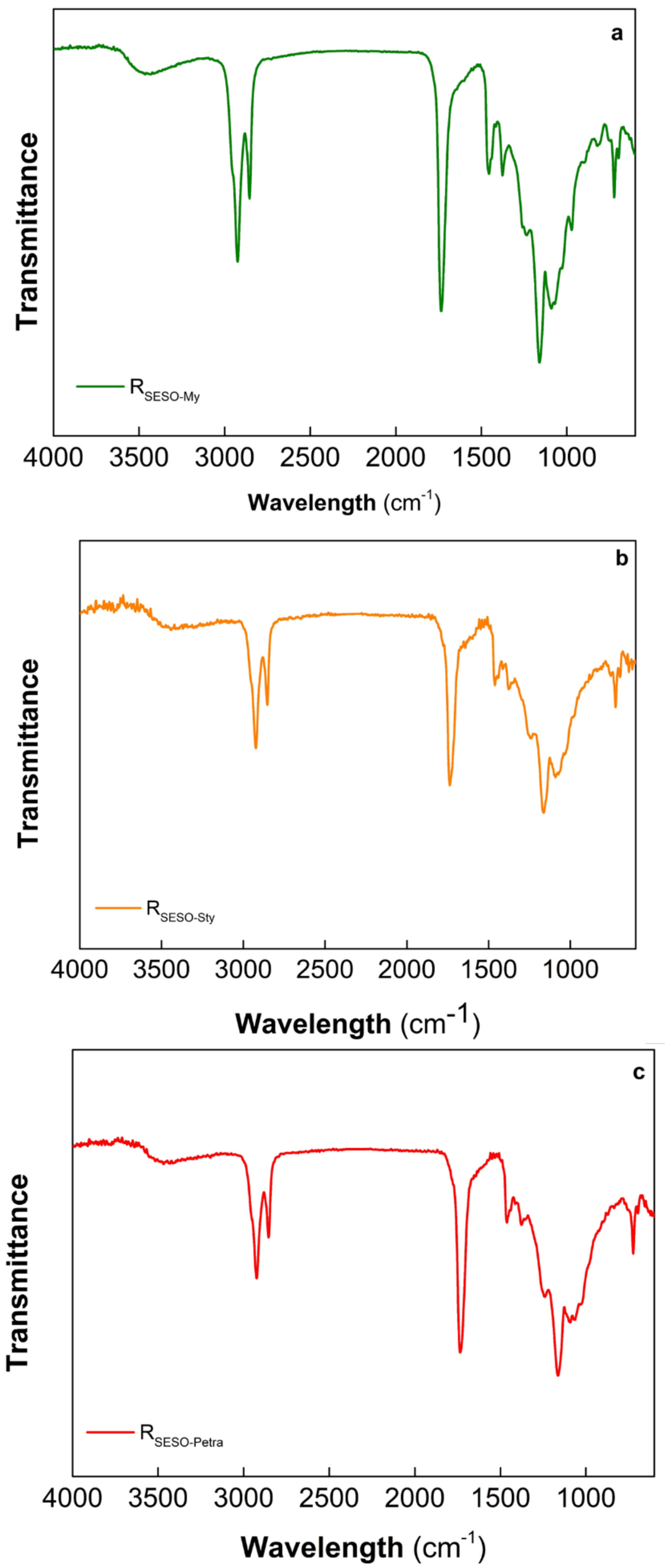

Figure S11. FTIR of resins prepared from SESO: (a) $R_{SESO-MY}$, (b) $R_{SESO-STY}$ and (c) $R_{SESO-PETRA}$

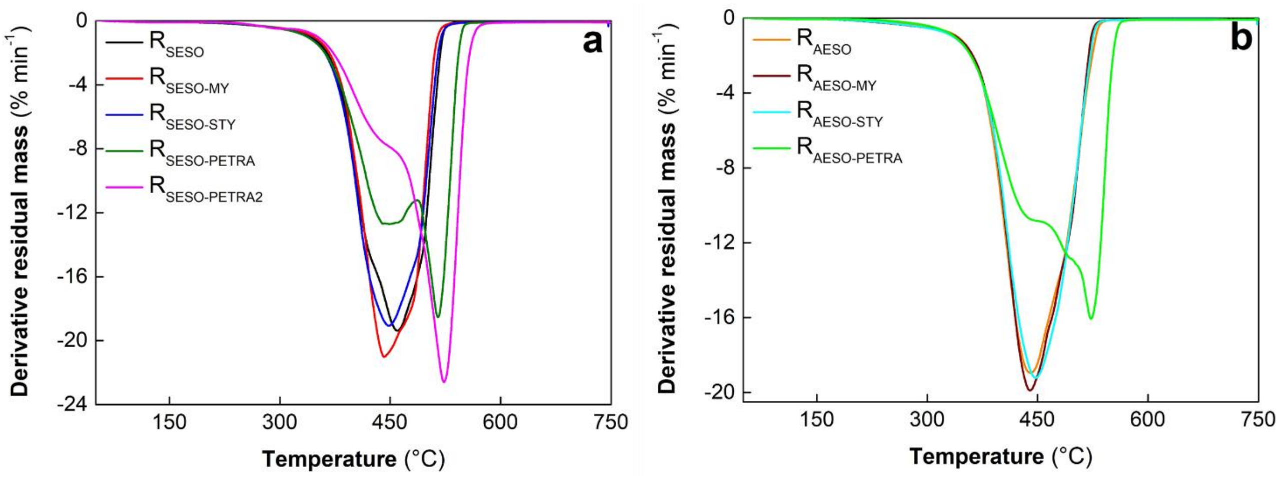

Figure S12. DTG curves of resins based on (a) SESO and (b) AESO
